# The Analgesic Effect of Morphine on Peripheral Opioid Receptors: An Experimental Research

**DOI:** 10.2478/jccm-2024-0042

**Published:** 2024-10-31

**Authors:** Nader-Mugurel Jafal, Smaranda Stoleru, Aurelian Zugravu, Carmen Orban, Mihai Popescu, Ruxandra Cristina Marin, Ion-Gigel Fulga

**Affiliations:** “Carol Davila” University of Medicine and Pharmacy, Bucharest, Romania

**Keywords:** morphine, carrageenan, analgesic effect, intraplantar administration, paw inflammation

## Abstract

Opioids represent one of the key pillars in postoperative pain management, but their use has been associated with a variety of serious side effects. Thus, it is crucial to investigate the timing and course of opioid administration in order to ensure a best efficacy to side-effect profile. The aim of our article was to investigate the analgesic effects of locally administered morphine sulfate (intraplantar) in a carrageenan-induced inflammation model in rats. After carrageenan administration, the rats were divided into 10 equal groups and were injected with either morphine 5 mg/kg or 0.9% saline solution at different time intervals, depending on the assigned group. The analgesic effect was assessed through thermal stimulation. Our results showed that paw withdrawal time was significantly higher in rats treated with morphine compared to those in the control group 9.18 ± 3.38 compared to 5.14 ± 2.21 seconds, p=0.012). However, differences were more pronounced at certain time intervals post-carrageenan administration (at 180 minutes compared to 360 minutes, p=0.003 and at 180 minutes compare to 1440 minutes p<0.001), indicating that efficacy varies depending on the timing of treatment. In conclusion, our findings support the hypothesis that locally administered morphine may alleviate pain under inflammatory conditions and underscores the importance of considering treatment timing when evaluating the analgesic effect.

## Introduction

Opioids constitute a class of pharmacological compounds that play a crucial role in pain management. However, despite being among the most potent and versatile analgesics currently available, our understanding of pharmacokinetics and pharmacodynamic properties remains incomplete, with various aspects of their interaction with the human body requiring further research. Although highly effective in alleviating severe pain, the widespread use of opioids is limited by the high incidence of serious side effects including respiratory depression, dependency, tolerance, nausea, and constipation [1]. These negative consequences can be severe and pose significant risks to patient health. Additionally, long-term opioid use may negatively impact quality of life and daily functioning [2, 3].

In the late 1980s, studies began to highlight that the action of opioids is not limited solely to opioid receptors in the brain and spinal cord, but also affects peripheral sensory neurons [4]. Research has shown that intrinsic modulation of pain sensations can occur at the level of peripheral nerve endings as well [5, 6]. The peripheral analgesic effects of opioids have been more evident in pathophysiological conditions such as inflammation, tissue injuries, or neuropathy [7, 8]. During inflammation, the permeability of the blood-brain barrier increases, and the expression of opioid receptors on peripheral sensory neurons is upregulated. This facilitates the interaction of opioids with their receptors at the site of injury, enhancing their analgesic effects [9]. Tissue damage results in the release of inflammatory mediators and an increase in opioid receptors in the peripheral nervous system. This localized upregulation allows opioids to exert their pain-relieving effects more effectively in the injured area [10]. In neuropathic pain, changes in the nervous system, such as altered receptor expression and increased release of endogenous opioids, enhance the peripheral action of exogenous opioids. These changes make peripheral opioid receptors more responsive to analgesic treatment [11].

Considering the experiments conducted over time, we aimed to assess whether locally administered morphine generates a local analgesic effect and to determine the time point after the onset of inflammation at which this effect is most pronounced.

## Materials and Methods

The current study protocol was approved by the Ethics Committee of “Carol Davila” University of Medicine and Pharmacy, Bucharest, Romania (7590/22065) for studies involving animals, in conformity with 43/2014 Law regarding animal protection used in scientific purposes, with further completions and 86/609/CEE Directive from 24 November 1986 regarding acts with power of law and administrative acts of member states for animal protection used in experimental purposes and other scientific purposes.

### Animals

For this experiment 100 Wistar rats with an approximate average weight of 250 grams were used. The animals were obtained from the bio-base of Carol Davila University of Medicine and Pharmacy in Bucharest. Upon arrival, the rats were 6 weeks old, and they were allowed a one-week period for acclimatization and adaptation to the new location before the experiment began. The experiment took place over a two-day period, and upon its completion, the animals were humanely euthanized under general anesthesia. We specify that the experiment was conducted in accordance with the ethics guidelines for research on laboratory animals and with the approval of the Ethics Committee within the institution.

### Experimental Procedures

The animals were divided into 10 equal groups, with each rat housed in a separate cage with unlimited access to water and food throughout the study. The environmental conditions of the workspace where the rats were kept remained unchanged (light, temperature, humidity).

All rats were injected in the right paw with 0.15 ml of 1% carrageenan. After different time intervals depending on the group in which the rats were allocated, they received either morphine at 5 mg/kg body weight or 0.9% saline solution in the same volume as the morphine solution as follows:

– Group 1 received 0.9% saline solution intraplantar in the right paw immediately after carrageenan administration.
– Group 2 received morphine at 5 mg/kg body weight intraplantar in the right paw immediately after carrageenan administration.
– Group 3 received 0.9% saline solution intraplantar in the right paw 3 hours after carrageenan administration.
– Group 4 received morphine at 5 mg/kg body weight intraplantar in the right paw 3 hours after carrageenan administration.
– Group 5 received 0.9% saline solution intraplantar in the right paw 6 hours after carrageenan administration.
– Group 6 received morphine at 5 mg/kg body weight intraplantar in the right paw 6 hours after carrageenan administration.
– Group 7 received 0.9% saline solution intraplantar in the right paw 24 hours after carrageenan administration.
– Group 8 received morphine at 5 mg/kg body weight intraplantar in the right paw 24 hours after carrageenan administration.
– Group 9 received 0.9% saline solution intraplantar in the right paw 48 hours after carrageenan administration.
– Group 10 received morphine at 5 mg/kg body weight intraplantar in the right paw 48 hours after carrageenan administration.

For each group, the analgesic effect was assessed through thermal stimulation using the Hargreaves method, also known as the plantar test, which is a widely used technique for assessing thermal nociception in animal models, particularly rodents. This method measures the latency of a withdrawal response to a thermal stimulus applied to the plantar surface of the paw [12]. The Ugo Basile device (founded by Ugo Basile in Germonio, Italy) was used, which automatically records the time of limb withdrawal in response to painful thermal stimulation through infrared light.

It is noted that the animals were acclimatized to the working environment and to the specific cages of the apparatus in the days preceding substance administration.

For each subject, the assessment began 10 minutes after morphine or saline injection, and 5 assessments were conducted at 5-minute intervals, with minimal and maximal deviations eliminated.

### Statistical analysis

For statistical processing of the study data, IBM SPSS Statistics for Windows, Version 29.0 (30-day trial version) Armonk, NY: IBM Corp was used. Continuous variables were analyzed for normality and then expressed as mean ± standard deviation, median, minimum, and maximum. The Mann-Whitney U test was used to compare the mean values of the Right Paw Withdrawal Time variable between groups. The Kruskal-Wallis H test was used to compare the mean values of parameters between groups, considering that the variables have a non-normal distribution. A p-value <0.05 was considered statistically significant.

## Results

### Comparison of Withdrawal Time between Control and Morphine-Administered Groups

To highlight the local analgesic effect of morphine, we compared the control groups with the groups to which morphine was administered. In Table 1, the mean withdrawal times for both the control and experimental rats can be observed, depending on the time morphine was administered.

**Table 1.** Comparison of withdrawal times between the control and experimental groups depending on the time morphine was administered.

**Groups**	**N**	**Withdrawal time (s)**	**Statistic test**	
			**Mann-Whitney U**	**P value**
Group control 1 – 0.9% saline	10	5.742 ± 2.452	27.500	0.089
Group 2 – morphine 5 mg/kg	10	7.658 ± 2.253		

Group control 3 – 0.9% saline (3h)	9	5.146 ± 2.212	12.000	0.012^*^
Group 4 - morphine 5 mg/kg (3h)	9	9.187 ± 3.389		

Group control 5 – 0.9% saline (6 h)	10	3.539 ± 1.161	17.000	0.011^*^
Group 6 - morphine 5 mg/kg (6 h)	10	5.021 ± 1.427		

Group control 7 – 0.9% saline (24 h)	10	2.746 ± 0.873	23.000	0.043^*^
Group 8 - morphine 5 mg/kg (24 h)	10	3.429 ± 0,446		

Group control 9 – 0,9% saline (48 h)	10	2.980 ± 0.758	19.500	0.019^*^
Group 10 - morphine 5 mg/kg (48 h)	10	3.836 ± 0.662		

**Fig. 1.**
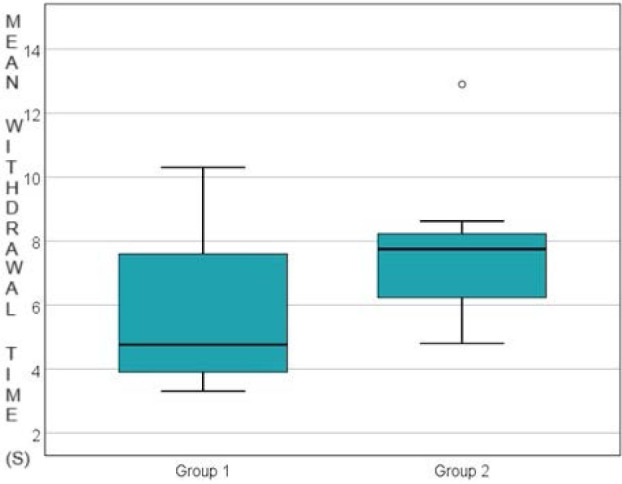
Comparison of the control group vs. morphine group immediately after carragenan administration

We observed no significant difference in mean withdrawal time immediately after carrageenan administration for the control and experimental groups (5.742 ± 2.452 vs. 7.658 ± 2.253, p=0.089). (Figure 1) Starting from the 3 hours interval we observed a significant longer withdrawal time in the experimental group compared to the control group that lasted up to the 48 hours interval for morphine administration after carrageenan injection (Table 1).

**Table 2.** Comparison of withdrawal time between the morphine administration groups

**Groups**	**N**	**Withdrawal time (s)**	**Statistic test**	
			**Mann-Whitney U**	**p value**
Group 2 – morphine 5 mg/kg	10	7.658 ± 2.253	30.000	0.243
Group 4 - morphine 5 mg/kg (3h)	9	9.187 ± 3.389		

Group 2 – morphine 5 mg/kg	9	7.658 ± 2.253	12.500	0.003^*^
Group 6 - morphine 5 mg/kg (6 h)	9	5.021 ± 1.427		

Group 2 – morphine 5 mg/kg	10	7.658 ± 2.253	0.000	<0.001^*^
Group 8 - morphine 5 mg/kg (24 h)	10	3.429 ± 0.446		

Group 2 – morphine 5 mg/kg	10	7.658 ± 2.253	1.000	<0.001^*^
Group 10 - morphine 5 mg/kg (48 h)	10	3.836 ± 0.662		

Group 4 - morphine 5 mg/kg (3h)	9	9.187 ± 3.389	11.000	0.004
Group 6 - morphine 5 mg/kg (6 h)	10	5.021 ± 1.427		

Group 4 - morphine 5 mg/kg (3h)	9	9.187 ± 3.389	2.000	<0.001^*^
Group 8 - morphine 5 mg/kg (24 h)	10	3.429 ± 0.446		

Group 4 - morphine 5 mg/kg (3h)	9	9.187 ± 3.389	3.500	<0.001^*^
Group 10 - morphine 5 mg/kg (48 h)	10	3.836 ± 0.662		

Group 6 - morphine 5 mg/kg (6 h)	10	5.021 ± 1.427	8.000	0.001^*^
Group 8 - morphine 5 mg/kg (24 h)	10	3.429 ± 0.446		

Group 6 - morphine 5 mg/kg (6 h)	10	5.021 ± 1.427	20.500	0.023
Group 10 - morphine 5 mg/kg (48 h)	10	3.836 ± 0.662		

Group 8 - morphine 5 mg/kg (24 h)	10	3.429 ± 0.446	32.500	0.190
Group 10 - morphine 5 mg/kg (48 h)	10	3.836 ± 0.662		

**Fig. 2.**
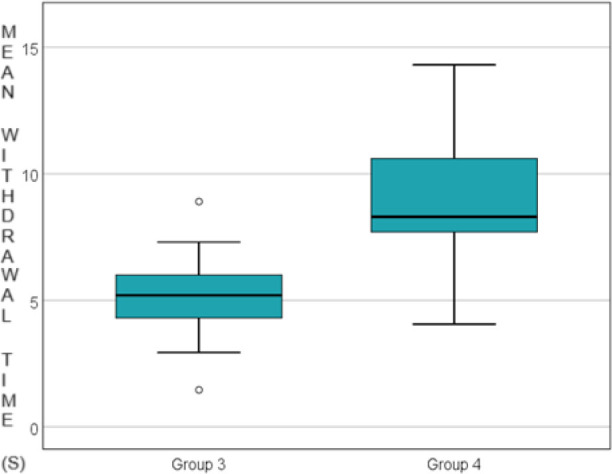
Comparison of the control group vs. morphine group 3 hours after carragenan administration

Regarding the control group and the experimental group in which morphine was administered 3 hours after carrageenan, the paw withdrawal time was significantly longer in the experimental group (5.146 ± 2.212 vs 9.187 ± 3.389 p= 0.012) (Figure 2).

**Fig. 3.**
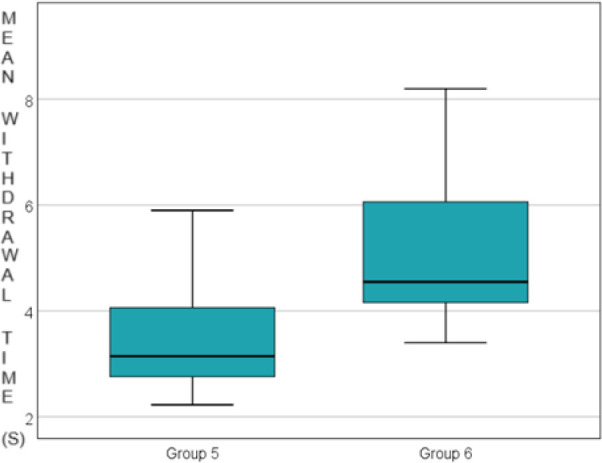
Comparison of the control group vs. morphine group 6 hours after carragenan administration

**Fig. 4.**
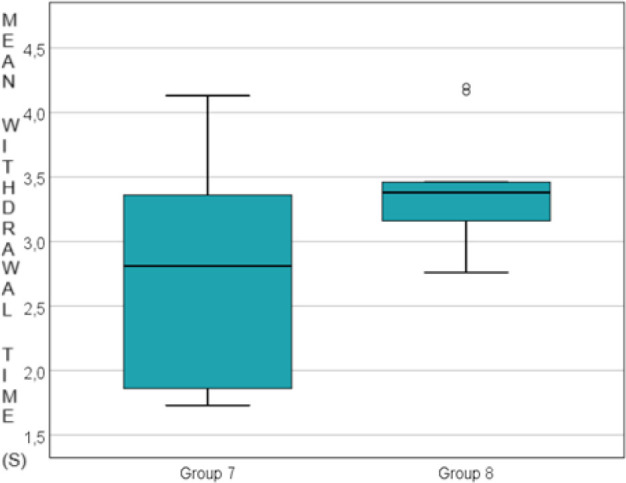
Comparison of the control group vs. morphine group 24 hours after carragenan administration

**Fig. 5.**
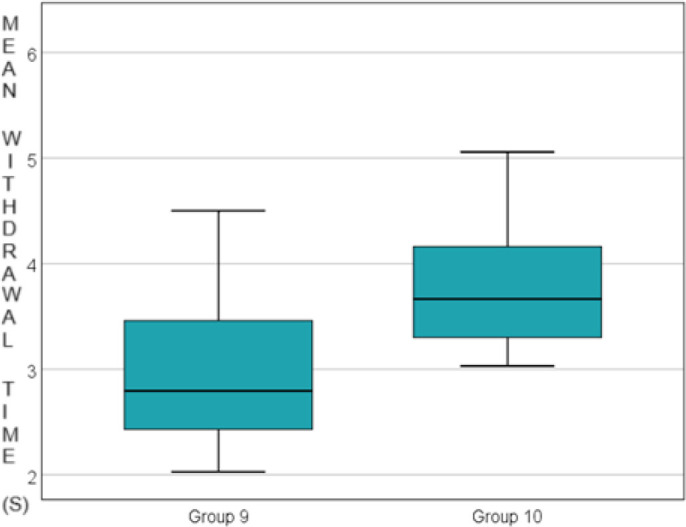
Comparison of the control group vs. morphine group 48 hours after carragenan administration

Additionally, a statistically significant increase in paw withdrawal time was observed between the control groups and the corresponding experimental groups (at 6, 24, and 48 hours) across all experimental groups: 3.539 ± 1.161 vs. 5.021 ± 1.427 seconds at 6 hours, p=0.011, 2.746 ± 0.873 vs. 3.429 ± 0.446 seconds at 24 hours, p=0.043 and 2.980 ± 0.758 vs. 3.836 ± 0.662 seconds at 48 hours, p=0.019. Data are presented in Figure 3, Figure 4 and Figure 5.

### Comparison of withdrawal time between the morphine administration groups

**Fig. 6.**
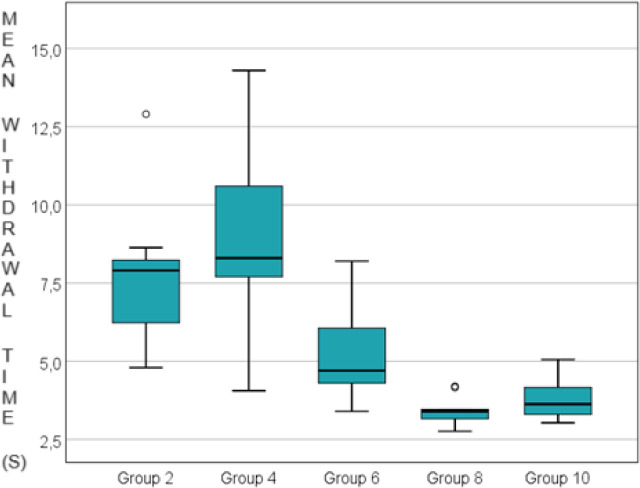
The mean withdrawal time of paws in the 5 experimental groups

To determine significant differences among the five groups regarding withdrawal times, pairwise comparisons were conducted using the Mann-Whitney U test, and the significance threshold was adjusted based on the number of comparisons (ten in our case), resulting in p=0.05/10=0.005. The results obtained, adjusted for the corrected significance threshold based on the number of comparisons, were as follows (Table 2). We observed that the withdrawal time was significantly higher in group 2 compared to group 6 (p=0.003), group 8 (p<0.001) and group 10 (p<0.001). Also, withdrawal time was significantly higher in group 4 compared to group 8 (p=0.004) and group 10 (p<0.001) and in group 6 compared to group 8 (p=0.001). Thus, withdrawal times immediately after carrageenan administration and at 3 hours post-carrageenan administration significantly differ from those at 6 hours, 24 hours, and 48 hours post-carrageenan administration (Figure 6).

We also wanted to see if the mean differences between the 5 sets of control-test pairs show statistically significant differences. The difference between the mean paw withdrawal times for the control-test pair, where morphine was administered immediately after carrageenan, is 1.916 ± 3.143. For the control-test pair at 3 hours, the mean difference between the groups was 4.041 ± 3.874, at 6 hours it was 1.482 ± 1.361, at 24 hours it was 0.683 ± 0.627, and finally at 48 hours it was 0.856 ± 0.543. The p-value = 0.004 suggests that there are statistically significant differences between at least 2 of the compared pairs. It should also be noted that the largest difference was in the control-test pair that received morphine at 3 hours, where the mean difference was 4.041 ± 3.874.

## Discussion

Initially, it was believed that opioids exerted analgesic effects exclusively through actions within the central nervous system [13]. However, in recent decades, evidence has emerged suggesting that opioid antinociception can be initiated by activating opioid receptors located outside the central nervous system [14]. One of the earliest findings supports the notion that morphine could elicit analgesic effects upon local application to painful peripheral areas. Since then, numerous clinical and experimental reports have been published confirming similar observations [15].

Peripheral opioid receptors are located outside the central nervous system that interact with opioid substances. They are classified into peripheral mu (MOR), delta (DOR), and kappa (KOR) receptors [16]. Their main role is to modulate sensations of pain and inflammation outside the brain and spinal cord. Activation of peripheral opioid receptors can produce analgesic effects, reducing the sensation of pain and inflammation in those areas [17]. Although initial attempts to demonstrate peripheral opioid analgesia in unaffected tissues yielded controversial results, subsequent studies conducted by Stein and his research team in pathological pain models were more successful and showed that the local injection of low, systemically inactive doses of agonists produced dose-dependent, stereospecific, and reversible analgesia through selective opioid antagonists [18]. Additionally, antinociception has been demonstrated in models of neuropathic, visceral, incisional, thermal, bone, and cancer pain. According to in vitro studies, the co-administration of agonists may act synergistically [19].

The peripheral analgesic effects of opioids are particularly evident in inflamed tissue. Under such conditions, the synthesis and expression of opioid receptors in dorsal root ganglia are increased [20, 21]. According to a review by researcher Stein, interleukin IL-4 and activator protein-1 are involved in stimulating the transcription of opioid receptors [22]. Furthermore, inflammation in peripheral tissues can amplify the synthesis and expression of these receptors, facilitating their axonal transport and increasing agonistic efficacy at nerve terminals. Inflammation may also increase the number of sensory nerve terminals and disrupt the perineural barrier, thereby promoting the access of opioid agonists to their receptors [23]. Clinical studies indicate that the application of opioid agonists to un-injured nerves is not reliably effective in producing analgesic effects, suggesting that inflammation promotes the accessibility and efficient coupling of opioid receptors in primary afferent neurons [24, 25]. Additionally, the secretion of endogenous opioid ligands in inflamed tissues may contribute to synergistic interactions at peripheral opioid receptors [26].

Our study showed that the limb withdrawal time was significantly higher in rats treated with intraplantar morphine compared to those in the control group. The study observed variations in the effectiveness of morphine depending on the timing of treatment post-carrageenan administration. We must consider that in the carrageenan-induced inflammation model, the peak level of inflammation is reached 3–5 hours after administration [27]. Carrageenan-induced local inflammation exhibits an acute phase (within the first 3 hours) dominated by the release of pro-inflammatory mediators such as histamine, serotonin, and bradykinin [28]. This is followed by an intermediate phase lasting up to 6 hours, where leukocyte infiltration and the release of large amounts of prostaglandins predominate. After 24 hours, the inflammation is characterized by a subacute phase with intense cellular infiltration [29].

These changes likely explain why the analgesic effect is most pronounced 3 hours after carrageenan administration, a time when the inflammation is actively progressing with the release of prostaglandins and other inflammatory cytokines. Additionally, this phase may also be marked by increased sensitivity of peripheral neurons due to the higher expression of opioid receptors at this stage of inflammation [30].

This highlights the importance of considering the temporal aspect of treatment in assessing analgesic efficacy. While the study focused on evaluating the analgesic effect of morphine, future research could delve into the underlying mechanisms responsible for its efficacy in inflammatory pain.

However, it is important to acknowledge certain limitations of the study. While the carrageenan-induced inflammation model is widely used and well-characterized, it may not fully replicate the complexity of clinical inflammatory pain conditions. Moreover, the study primarily focused on assessing the analgesic effects of morphine and did not delve into the underlying molecular mechanisms involved.

## Conclusions

Locally administered morphine (intraplantar) demonstrates a significant analgesic effect in a carrageenan-induced inflammation model in rats, with its efficacy varying depending on the timing of administration following inflammation induction. The most pronounced analgesic effects are observed when morphine is administered immediately after carrageenan injection and at 3 hours post-administration, a period corresponding to the peak of inflammation characterized by the release of prostaglandins and other inflammatory cytokines. These findings suggest that locally administered morphine could be a promising option for pain relief in inflammatory conditions, with the timing of administration being crucial to maximizing its effectiveness. The enhanced sensitivity of peripheral neurons due to increased opioid receptor expression during this phase further supports the importance of precise timing in achieving optimal analgesic outcomes.
